# Impact of central adjudication of the score on the modified Rankin Scale in an international, randomized, acute stroke trial

**DOI:** 10.1177/23969873251320207

**Published:** 2025-02-19

**Authors:** Wouter M Sluis, Jeroen C de Jonge, Hendrik Reinink, Alastair Wilson, Lisa J Woodhouse, Jesse Dawson, Kennedy R Lees, Philip M Bath, Hendrik Bart van der Worp

**Affiliations:** 1 Department of Neurology and Neurosurgery, UMC Utrecht Brain Center, University Medical Center Utrecht, Utrecht University, Utrecht, The Netherlands; 2 Department of Neurology, Onze Lieve Vrouwe Gasthuis, Amsterdam, The Netherlands; 3 Department of Neurology, Spaarne Gasthuis, Haarlem, The Netherlands; 4 School of Medicine, Dentistry and Nursing, University of Glasgow, Glasgow, UK; 5 Stroke Trials Unit, Mental Health & Clinical Neuroscience, University of Nottingham, Nottingham, UK; 6 Institute of Cardiovascular and Medical Sciences, College of Medical, Veterinary and Life Sciences, University of Glasgow, Glasgow, UK

**Keywords:** Ischemic stroke, modified Rankin Scale, intracerebral hemorrhage, interobserver variability, aspiration, pneumonia, randomized trial, PRECIOUS

## Abstract

**Introduction:**

The modified Rankin Scale (mRS) is the most frequently used primary outcome measure in acute stroke research despite significant interobserver variability in assigning grades. We assessed the use of central blinded adjudication of the mRS based on a video recording of an interview in the PRECIOUS trial.

**Patients and methods:**

PRECIOUS was an international, randomized, open-label, clinical trial with blinded outcome assessment of preventive treatment with metoclopramide, paracetamol, and ceftriaxone in elderly patients with acute stroke. Trained local investigators interviewed patients or their representatives and graded functional outcome at 90 days after stroke with the mRS. In each participating country, a video recording of the interview was assessed by three blinded, independent adjudicators. The primary outcome of the present substudy was interobserver agreement between the local mRS score and the median score of the three central adjudicators for patients alive at 90 days, assessed with Cohen’s kappa and quadratic weighted kappa statistics. The difference between treatment effect estimates based on local and central adjudication was a secondary outcome.

**Results:**

Of 1493 patients enrolled in PRECIOUS, 1471 were included in this analysis. At 90 days, 1117 patients (75.9%) were alive and had both a central and local assessment; 28 participants did not have a central mRS score. Interobserver agreement was seen in 829 (74.2%) patients and was substantial (kappa of 0.68; 95% CI 0.65–0.71). Disagreement occurred more often in patients with a central mRS score of 0–2 (OR 2.24; 95% CI 1.14–4.24). Treatment effects were neutral for all three study drugs and did not differ between central and local adjudication.

**Discussion and conclusion:**

Central adjudication of the mRS based on a video recording is feasible in a large international, randomized stroke trial. This ensures blinding of the outcome assessment. In this neutral trial, the impact of central adjudication on the precision of effect size estimates could not be assessed.

## Graphical abstract

**Graphical abstract:**
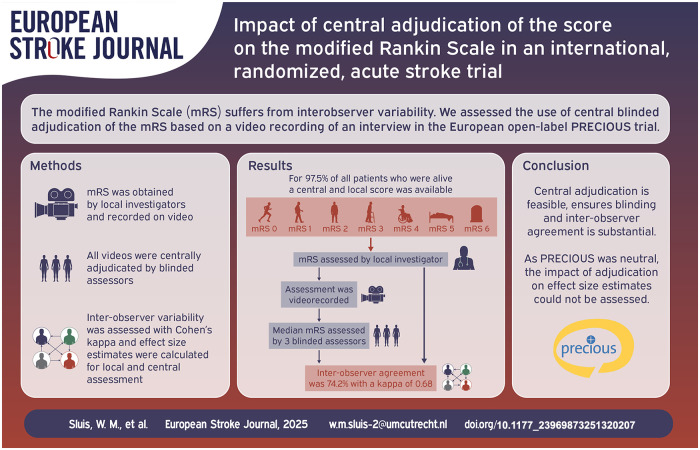

## Introduction

The modified Rankin scale (mRS) is the most frequently used primary outcome measure in acute stroke research.^1^ It is an ordinal scale with six categories ranging from zero through five, categorizing the degree of disability or dependence in the daily activities. In currently used versions, a seventh category is added to signify death.^2^ Despite its wide-spread use as a primary outcome measure, the reliability of the mRS is hampered by significant inter-observer variability,^3–6^ which may reduce statistical power and negatively influence the validity of study results, especially in studies without proper blinding of the adjudicator.^7^

Central outcome adjudication of videoed recordings of the mRS by independent experts blinded to treatment allocation has been proposed to improve the reliability and precision of treatment estimates by limiting detection bias, reducing random or systematic errors, benefiting from expert opinion and experience of the raters, and to guarantee blinding. As it facilitates scoring of a single assessment by several raters with the possibility to combine these to control inter-observer variability, central outcome adjudication may enhance statistical power and permit smaller sample sizes.^8,9^ A systematic review and meta-analysis of randomized stroke trials found no evidence so far that this process has any impact on trial conclusions, but the mRS was the primary outcome in just one of the included trials.^10^ In two other, open-label, randomized trials of endovascular thrombectomy (EVT) for acute ischemic stroke in a single country, central adjudicators disagreed with the locally allocated mRS score in 13% and 11% of the cases, but this was not associated with material differences in estimates of treatment effect.^11,12^ In one of these trials central adjudication was based on a written report of the mRS interview,^12^ and in the other on an audio or video recording of the interview.^11^ These trials have generally been conducted within experienced centers, where the mRS is routinely used by staff. The value of central adjudication of the mRS in larger, international, open-label stroke trials conducted in less experienced settings is uncertain.

We assessed inter-observer variability and impact on treatment effect estimates of central mRS adjudication based on a video recording in the European, open-label, randomized PREvention of Complications to Improve OUtcome in elderly patients with acute Stroke (PRECIOUS) trial.^13^

## Methods

### Study protocol and population

We included patients enrolled in PRECIOUS, a European, multi-center, 3 × 2-factorial, randomized, controlled, open-label clinical trial with blinded outcome assessment of preventive use of metoclopramide versus no metoclopramide, ceftriaxone versus no ceftriaxone, and paracetamol versus no paracetamol for 4 days in patients aged 66 years or older with acute ischemic stroke or intracerebral hemorrhage and a score on the National Institutes of Health Stroke Scale (NIHSS) ⩾6 (ISRCTN82217627).^13–15^ Exclusion criteria for PRECIOUS included an active infection requiring antibiotic treatment, a pre-stroke score on the mRS ⩾4, and death appearing imminent at screening. For the interobserver analyses of the present substudy, we excluded patients who had died before the outcome assessment at 90 days and those for whom a central or local mRS assessment was not available. The trial was approved by the central medical ethics committee of the University Medical Center Utrecht on 3 February 2016 and by national or local research ethics committees in all participating countries. Patients, their legal representatives or independent physicians provided written informed consent.

### Data collection

The primary outcome in PRECIOUS was the score on the mRS at 90 (±14) days after randomization. Assessment was done by local investigators who had successfully completed an online training and certification provided by the central study team.^16^ The mRS interview with the patient or caregiver by the local investigator was recorded on video and uploaded to a secure server where these videos were assigned to three independent adjudicators from the country the video was recorded in. These adjudicators were also trained, certified and blinded to treatment allocation. During the COVID-19 pandemic, an audio recording of the interview was also allowed if a video recording was not possible. The median of the three central scores on the mRS was the primary outcome in PRECIOUS.

### Outcomes

The primary outcome of the current study was interobserver agreement between the local and central adjudicators of the mRS across all participating countries. Secondary outcomes were (1) interobserver agreement between the local and central adjudicators of the mRS in each of the participating countries; (2) predictors of disagreement between local and central assessment; and (3) differences in treatment effect estimates between local and central assessment.

### Statistical analysis

Interrater concordance between central and local adjudicators across all countries was evaluated by displaying crude percentages of disagreement between local and central assessment, visualized in a cross tabulation. For the central assessment, the median of the three scores on the mRS was used. Inter-observer variability was assessed with Cohen’s kappa statistics.^17^ Interpretation of kappa values was as follows: values less than 0.00 indicate poor agreement, 0.00–0.20 slight agreement, 0.21–0.40 fair agreement, 0.41–0.60 moderate agreement, 0.61–0.80 substantial agreement, and 0.81–1.00 almost perfect agreement.^18^ Because the mRS is an ordinal rather than a linear scale, we also calculated quadratic weighted kappas.^19^ Crude percentages of agreement and disagreement were also displayed per country. Comparisons between patients with disagreement on the mRS and those with agreement were made using χ^2^ or Student’s *t*-test, where appropriate. Afterwards, variables with a *p* value less than 0.15 in the univariable model were included in a multivariable logistic regression analysis to identify independent determinants of disagreement.

The effects of preventive ceftriaxone, metoclopramide, and paracetamol on the mRS score at 90 days as adjudicated locally or centrally were analyzed with adjusted ordinal logistic regression and expressed as adjusted common odds ratios (OR) with 95% confidence intervals (CI). The effect of treatment on the rate of death or dependency (mRS 3–6) was assessed with adjusted logistic regression. Analyses were adjusted for stratification (country), minimization (age, sex, stroke type, stroke severity, diabetes), baseline prognostic factors (premorbid mRS, atrial fibrillation, reperfusion treatment [alteplase and/or thrombectomy], time from onset to randomization), and treatment allocation in the other two strata of the trial. All statistical analyses were performed with R studio statistical software (version 1.3.1056) or SAS version 9.4 (SAS institute, Cary, NC, United States).

## Results

**Figure 1.**
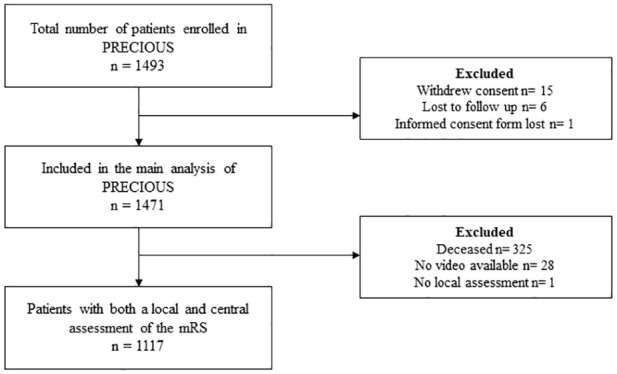
Flowchart of patient inclusion.

Through April 2016 and June 2022, 1493 patients from 68 sites in nine European countries were included in PRECIOUS. After excluding patients who were lost to follow-up, withdrew consent or whose informed consent form was lost, 1471 patients were included in this analysis (Figure 1).

At 90 days, 1146 patients (77.9%) were alive and for 1118 of these (97.6%) a video or audio recording was available and centrally adjudicated. For one patient, a video was uploaded and centrally adjudicated, but no local assessment was provided in the electronic case report form. For these reasons, 1117 (97.5%) patients with both a local and a central assessment were included in the primary analysis of the present study.

**Figure 2.**
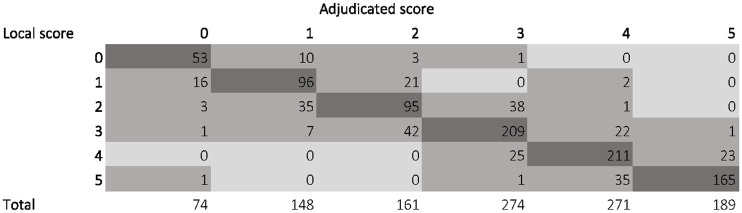
Distribution of local and median central assessment of the mRS score. mRS: modified Rankin Scale.

The median mRS score of the central adjudicators matched with the mRS score of the local adjudicators in 829 (74.2%) patients. The central score was higher than the local score in 122 patients (10.9%) and lower in 166 (14.9%). The distribution of local and central assessment of the mRS is shown in Figure 2.

After correction for chance, inter-observer agreement was substantial (kappa of 0.68; 95% CI 0.65–0.71) through near-perfect (quadratic weighted kappa of 0.92; 95% CI 0.92–0.92). The three central adjudicators were in unanimous agreement among themselves for 613 patients (54.9%) and this extended to include the local adjudicators for 524 patients (46.9%). Conversely, there was complete disagreement among the central adjudicators for 43 patients (3.8%) and in a single one of these (0.1%), the local rater had proposed a fourth score. There was no difference in misclassification rates between countries. (**Supplemental Table 1**).

Patients with disagreement between central and local adjudicators were more often graded as functionally independent by the central adjudicators (mRS 0–2; 48.3% of patients in whom disagreement occurred vs 29.4%, *p* < 0.001), mainly driven by patients who had a score of 1 (18.1% vs 11.6%, *p* = 0.007) or 2 (22.9% vs 11.5%, *p* < 0.001) and less often had a score of 5 on the mRS (8.3% vs 19.9%, *p* < 0.001). Further characteristics were comparable (Tables 1 and 2).

**Table 1.** Baseline and clinical characteristics of patients with and without agreement between local and central adjudicators.

Characteristics^a^	Agreement (*n* = 829)	Disagreement (*n* = 288)	*p*-Value^b^
Demographics			
Age, years (median; IQR)	78.0 (73.0–84.0)	78.0 (73.0–84.0)	0.473
Sex (female)	423 (51.0)	130 (45.1)	0.098
Clinical characteristics			
Pre-stroke mRS ⩾ 3	113 (13.6)	35 (12.2)	0.592
NIHSS (median; IQR)	11.0 (7.0–15.0)	10.0 (7.0–14.0)	0.056
Final clinical diagnosis			0.959
Ischemic stroke	702 (84.7)	243 (84.4)	
Intracerebral hemorrhage	114 (13.8)	41 (14.2)	
Stroke mimic	13 (1.6)	4 (1.4)	
Stroke location			0.439
Right hemisphere	257 (46.6)	94 (50.3)	
Left hemisphere	273 (49.5)	89 (47.6)	
Infratentorial	21 (3.8)	4 (2.1)	

IQR: interquartile range; mRS: modified Rankin Scale; NIHSS: National Institutes of Health Stroke Scale; SD: standard deviation.

a All numbers are *n* (%) unless stated otherwise.

b *p*-Values indicate the statistical differences in clinical and demographic characteristics between patients in whom central and local adjudicators agreed on the mRS score versus those in whom there was disagreement.

**Table 2.** Agreement or disagreement on modified Rankin Scale between local and central adjudicators.

mRS score at 90days^a^	Agreement
mRS 0 (*n* = 74)	53 (71.6)
mRS 1 (*n* = 148)	96 (64.9)
mRS 2 (*n* = 161)	95 (59.0)
mRS 3 (*n* = 274)	209 (76.3)
mRS 4 (*n* = 271)	211 (77.9)
mRS 5 (*n* = 189)	165 (87.3)
mRS 0–2 (*n* = 383)	244 (63.7)

mRS: modified Rankin Scale.

a Median mRS score as adjudicated centrally. All numbers are *n* (%) unless stated otherwise.

Only a central median mRS score of 2 was a predictor of disagreement (OR 2.42; 95% CI 1.14–4.24) between central and local adjudicators.

**Figure 3.**
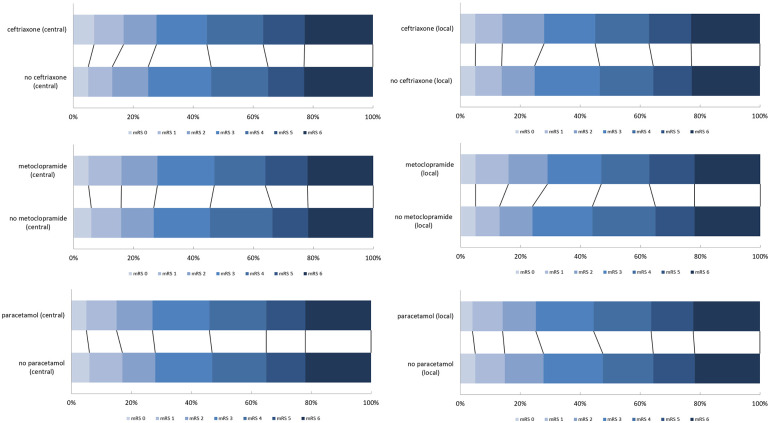
Distribution of the mRS scores based on local and central assessment for the paracetamol, metoclopramide and ceftriaxone stratums. mRS: modified Rankin Scale.

Treatment effect estimates for metoclopramide, ceftriaxone, or paracetamol based on local and central mRS adjudication were comparable when analyzed with ordinal logistic regression (Figure 3 and Table 3).

**Table 3.** Treatment effect estimates for a worse outcome obtained with ordinal logistic regression, based on local assessment versus central assessment of the score on the mRS at 90 days.

Treatment	acOR	95% CI
Ceftriaxone (local)	1.00	0.78–1.29
Ceftriaxone (central)	0.99	0.77–1.27
Paracetamol (local)	1.15	0.93–1.42
Paracetamol (central)	1.19	0.96–1.47
Metoclopramide (local)	0.99	0.80–1.24
Metoclopramide (central)	1.01	0.81–1.25

acOR: adjusted common odds ratio; CI: confidence interval.

The effects of treatment on the rate of death or dependency were numerically larger when based on local rather than central adjudication, but no effect estimate was statistically significant (Table 4).

**Table 4.** Treatment effect estimates obtained with a dichotomized analysis (mRS 0–2 vs 3–6), based on local assessment versus central assessment of the score on the mRS at 90 days.

Treatment	aOR	95% CI
Ceftriaxone (local)	0.83	0.58–1.18
Ceftriaxone (central)	0.91	0.64–1.29
Paracetamol (local)	1.19	0.88–1.61
Paracetamol (central)	1.15	0.85–1.55
Metoclopramide (local)	0.73	0.54–1.00
Metoclopramide (central)	0.84	0.62–1.14

aOR: adjusted odds ratio; CI: confidence interval.

## Discussion

In this large, randomized stroke trial that was performed in nine European countries, central adjudication of the mRS at 90 days based on a video or audio recording could be completed successfully in 98% of the surviving patients. Agreement between local and central adjudication of the mRS was substantial, and treatment effect estimates of the three interventions tested were similarly neutral based on either local or central assessment.

When compared to the randomized, open-label stroke trials MR CLEAN and REVASCAT, performed in a single country, the rate of disagreement between local and central assessment of the mRS was higher in our study: 25.8% versus 10.6% and 13.2%, respectively.^11,12^ In MR CLEAN, a structured interview with the patient or caregiver was performed by a single, experienced investigator not formally blinded to treatment allocation. This investigator then wrote a blinded report which was centrally adjudicated by a committee, consisting of five experienced vascular neurologists. This may leave less room for differences in interpretation than a video recording of a full interview, in which discrepancies with spoken answers may be evident, for example when a patient with obvious neglect claims to have no functional deficit. In REVASCAT a structured interview by a local neurologist was recorded on video or audiotape and centrally adjudicated by a single neurologist blinded to treatment allocation. This controls for potential bias and inter-observer variability, as well as ensuring that the rater is trained and experienced, but does not control for intra-observer variability. A possible explanation for the higher rate of disagreement in PRECIOUS is the larger number of central adjudicators, leaving more room for inter-observer variability, instead of a small group of central experienced adjudicators. Our rate of disagreement was however lower than that in a previous review of inter-observer variability assessments in stroke studies (37%).^20^

Our finding that the highest rates of disagreement were seen in patients who were centrally adjudicated as independent of others is comparable with a previous study.^21^ The quadratic weighted kappa of 0.92 in our study is comparable with that of REVASCAT (0.92) and corresponds well with the systematic review. No unweighted kappa was calculated in REVASCAT.^11^ In MR CLEAN, no kappa or weighted kappa was calculated.^12^

In line with MR CLEAN, there was no difference in treatment effect estimates when based on central or local mRS adjudication.^12^ This may be explained in part by the use of an ordinal shift analysis for the primary outcome, in which misclassification has a smaller impact than with dichotomized comparisons.^22^ In REVASCAT local assessment was associated with a slightly larger benefit of treatment than central assessment (OR 1.93 vs 1.71), but the sample size was small and confidence intervals were comparable.^11^ In a meta-analysis of 15 randomized stroke trials assessing either stroke prevention or acute treatment of stroke, there was no difference in effect estimates based on central or local assessment of the primary outcome, being recurrent stroke in eight trials, a composite event including stroke in six, and the mRS in one.^10^

We observed no difference in treatment effect estimates for metoclopramide, ceftriaxone, or paracetamol based on local and central mRS adjudication when analyzed with ordinal logistic regression. Central, blinded outcome adjudication could increase the precision of the effect estimates of an open-label clinical trial and therefore detect small effects that would have been missed by statistical noise from site-to-site variation in local outcome adjudication. This is of importance, as even a modest local differential misclassification has been shown to alter primary trial results, especially for a dichotomized outcome.^23^ Unfortunately, due to the neutral results of PRECIOUS we could not assess this potential benefit of central adjudication.

A limitation of the present study is the involvement of a broader group of “central” adjudicators in different countries, in contrast to smaller teams of central adjudicators in previous studies. Outcomes were assessed centrally in each of the nine participating countries, to meet the requirement that the assessor spoke the same language as used in the interview. PRECIOUS was performed in 67 academic and non-academic stroke centers to increase the external validity of the findings. For these reasons, the experience of the central and local adjudicators may have been less than that of adjudicators in previous studies, even though all adjudicators were trained and certified through the same standardized approach.^16^

## Conclusions

Central adjudication of the mRS with a video recording in an international, large, pragmatic, open-label, randomized stroke trial is feasible. This ensures blinding of the primary outcome assessment and increases precision by multiple measurements. In PRECIOUS, central adjudication had no impact on trial interpretation but as treatment effects were all neutral, conclusions on the impact and added value of central adjudication could not be drawn.

## Supplemental Material

sj-pdf-1-eso-10.1177_23969873251320207 – Supplemental material for Impact of central adjudication of the score on the modified Rankin Scale in an international, randomized, acute stroke trial Supplemental material, sj-pdf-1-eso-10.1177_23969873251320207 for Impact of central adjudication of the score on the modified Rankin Scale in an international, randomized, acute stroke trial by Wouter M Sluis, Jeroen C de Jonge, Hendrik Reinink, Alastair Wilson, Lisa J Woodhouse, Jesse Dawson, Kennedy R Lees, Philip M Bath and Hendrik Bart van der Worp in European Stroke Journal
