# Supplementary material for: Impact of central adjudication of the score on the modified Rankin Scale in an international, randomized, acute stroke trial
Source: Eur Stroke J. 2025 Feb 19;10(3):961–7. doi: 10.1177/23969873251320207 (PMC11840822; doi:10.1177/23969873251320207)
Supplement: sj-pdf-1-eso-10.1177_23969873251320207 – Supplemental material for Impact of central adjudication of the score on the modified Rankin Scale in an international, randomized, acute stroke trial [file sj-pdf-1-eso-10.1177_23969873251320207.pdf]

<u>Supplemental material to</u>

Impact of blinded outcome adjudication of the modified Rankin Scale in a large Open-Label European randomized stroke trial. Data from the PRECIOUS trial

Wouter M Sluis, Jeroen C de Jonge, Hendrik Reinink, Alastair Wilson, Lisa J Woodhouse, Jesse Dawson, Kennedy R Lees, Philip M Bath, H Bart van der Worp for the PRECIOUS investigators

Table S1. Differences in misclassification between the central and local assessment per country.

**Country***	**Disagreement (n%)**	**κ (95%CI)**
Country A	75 (27.2)	0.66 (0.60-0.73)
Country B	78 (28.3)	0.65 (0.58-0.71)
Country C	44 (24.0)	0.69 (0.61-0.77)
Country D	35 (22.2)	0.71 (0.63-0.80)
Country E	11 (32.4)	0.60 (0.40-0.80)
Country F	11 (37.9)	0.48 (0.24-0.73)
Country G	17 (18.7)	0.77 (0.67-0.87)
Country H	17 (25.8)	0.68 (0.55-0.81)

***** one country not analysed due to both a very low number of inclusions and no misclassifications

PRECIOUS investigators

Bart van der Worp, Jeroen de Jonge, Wouter Sluis, Rik Reinink, Berber Zweedijk, University Medical Center Utrecht, Utrecht, the Netherlands. Diederik van de Beek, Willeke Westendorp, Amsterdam UMC, Amsterdam, the Netherlands; Henk Kerkhoff, Elles Zock, Corry Deuling, Albert Schweitzer Ziekenhuis, the Netherlands; Sebastiaan de Bruijn, Yvonne Drabbe-Coops, Amy Nijst, Haga ziekenhuis, the Hague, the Netherlands; Marieke Wermer, Ghislaine Holswilder, LUMC, Leiden, the Netherlands; Korne Jellema, Peggy Sorensen, Haaglanden Medical Center, the Hague, the Netherlands; Vincent Kwa, Sarah Godefrooij, OLVG, Amsterdam, the Netherlands; Ben Jansen, Esther Santegoets, St Elisabeth Ziekenhuis, Tilburg, the Netherlands; Tobien Schreuder, Tiny Sporken, Zuyderland Medical Center, Heerlen, the Netherlands; Sanne Zinkstok, Kitty Harrison, Tergooi Medical Center, Hilversum, the Netherlands; Walid Moudrous, Chantal van der Spoel, Arienne Verwijs-Bode, Maasstad ziekenhuis, Rotterdam, the Netherlands; Malcolm Macleod, Allan MacRaild, Royal Infirmary of Edinburgh, Edinburgh, UK; Ruth Davies, Jessica Teasdale, Arrowe Park Hospital, Wirral, UK; Anand Nair, Venetia Johnson, Calderdale Royal Hospital, Calderdale, UK; Dipankar Dutta, Matthew Robinson, Jill Greig, Gloucestershire Royal Hospital, Gloucester, UK; Rohan Pathansali, Deborah Ward, Jon Glass, Jonnie Aeron-Thomas, Myriam Aissa, Fong Kum Chan, Staci Conway, Beatrix Sari, Maria Tibaja, King’s College Hospital, London, UK; Martin Cooper, Inez Wynter, Sherwood Forest Hospital, Sutton in Ashfield, UK; Kailash Krishnan, Camille Hutchinson, Ben Jackson, Nottingham University Hospitals, Nottingham, UK; Khalid Rashed. Sarah Board, Yeovil District Hospital, Yeovil, UK; Louise Shaw, Suzanne Lucas, Joanne Avis, Telma Costa, Lauren Pearce, Royal United Hospital, Bath, UK; Alastair Wilson, Johann Selvarajah, Angela Welch, Shirley Mitchell, Queen Elisabeth University Hospital, Glasgow, UK; Jessica Redgrave, Emma Richards, Jo Howe, Royal Hallamshire Hospital, Sheffield, UK; Mary Joan Macleod, Janice Irvine, Vicky Taylor, Aberdeen Royal Infirmary, Aberdeen, UK; Philip Clatworthy, Kerry Smith, North Bristol NHS Trust, Bristol, UK; Vasileois Papavasileiou, Emelda Veraque, Dean Waugh, Leeds Teaching Hospitals NHS Trust, Leeds, UK; Vera Cvoro, Mandy Couser, Amanda McGrego, Victoria Hospital, Kirkcaldy, UK; Brian Clarke, Ghatala Rita, Cai Hua Sim, Sarah Stratton, St. George Hospital, London, UK; Omid Halse , Peter Wilding, Sheila Mashate, Vaishali Dave, Imperial College, London, UK; Usman Ghani, Faith Omoregie, Yates Kimberley, University Hospital Coventry and Warwickshire, Coventry, UK; Janika Korv, Tartu University Hospital, Tarty, Estonia; Katrin Antsov, Parnu Hospital, Parnu, Estonia; Katrin Gross-Paju, West Talinn Hospital, Tallinn, Estonia; Inga Kalju, Maarja Kaarlop, East Tallinn Hospital, Tallinn, Estonia; Gotz Thomalla, Hannes Appelbohm, Christoph Brosinski, University Medical Center Hamburg-Eppendorf, Hamburg, Germany; Gerhard Hamann, Michaela Vogel, Bezirkskrankenhaus Günzburg, Günzburg, Germany; Michael Rosenkranz, Stefan Boskamp, Albertinen-Krankenhaus Hamburg, Hamburg, Germany; Christoph Gumbinger, Peter Ringleb, Elisabeth Beyrle, Universitätsklinikum Heidelberg, Heidelberg, Germany; Georg Royl, Susanne Ribau, Universitätsklinikum Schleswig-Holstein, Lübeck, Germany; Sven Poli, Julia Zeller, Sonja Ruschitzka, Universitätsklinikum Tübingen, Tübingen, Germany; Susanne Müller, Andrea Schirmer, Universitätsklinikum Ulm, Ulm, Germany; George Ntaios, Efstathia Karagkiozi, Larissa University Hospital, Larissa, Greece; Sophie Vassilopoulou, Aeginition Hospital, Athens, Greece; Haralampos Milionis, Angelos Liontos, Ioannina University Hospital, Ioannina, Greece; Athanasios Protogerou, Stamatia Samara, Laiko Hospital, Athens, Greece; Efsthathios Manios, Efthalia Mitsikosta, Alexandra Hospital, Athens, Greece; Laszlo Csiba, Krisztina Buzás-Petrócz, Csilla Vér, University of Debrecen, Debrecen, Hungary; Dániel Bereczki, Andrea Kovacs,

Semmelweis University, Budapest, Hungary; Gábor Jakab, Uzsoki Hospital, Budapest, Hungary; Ferenc Nagy, Lõrinczy Ritta, Puskásné Emmer Mária, Jávorszky Ödön Hospital, Vác, Hungary; András Folyovich, Nadim Al-Muhanna, Szent János Hospital, Budapest, Hungary; László Szapáry, Eszter Jozifek, University of Pecs, Pecs, Hungary; Alfonso Ciccone, Giorgio Silvestrelli, Paola Danesi, Marco Russo, ASST di Mantova, Mantova, Italy; Nicola Gilberti, Spedali Civili, Brescia, Italy; Enrico Righetti, Ospedale Castiglione del Lago, Castiglione del Lago, Italy; Stefano Ricci, Maria Elena Mattace, Silvia Cenciarelli, Ospedale di Città di Castello, Città di Castello, Italy; Stefano Ricci, Ospedale Gubbio – Gualdo Tadino, Branca, Italy; Pietro Bassi, Ospedale San Giuseppe, Milano, Italy; Simona Marcheselli, IRCCS Istituto Clinico Humanitas, Rozzano, Italy; Alessia Giossi, ASST Cremona, Cremona, Italy; Paolo Candelaresi, Giovanna Servillo, Ospedale Antonio Cardarelli, Napoli, Italy; Eivind Berge, Anne Hege Aamodt, Oslo University Hospital, Oslo, Norway; Anne Gro Holtan, Notodden Sykehus, Notodden, Norway; Sameer Maini, Alesund Hospital, Alesund, Norway; Iwona Kurkowska, Michal Karlinski, Institute of Psychiatry and Neurology, Warsaw, Poland; Waldemar Fryze, Malgorzata Krzyzanowska, University of Gdansk, Gdansk, Poland; Waldemar Brola, Hospital Sw. Lukasza, Konskie, Poland; Piotr Sobolewski, Szpital Specjalistyczny Ducha Swietego, Sandomierz, Poland; Marta Bilik, Samodzielny Publiczny Specjalistyczny Szpital Zachodni im. św. Jana Pawła II, Grodzisk Mazowiecki, Poland.

CONSORT 2010 checklist of information to include when reporting a randomised trial*

Section/Topic	Item No	Checklist item	Reported on page No
**Title and abstract**			
	1a	Identification as a randomised trial in the title	1
	1b	Structured summary of trial design, methods, results, and conclusions (for specific guidance see CONSORT for abstracts)	3
**Introduction**			
Background and objectives	2a	Scientific background and explanation of rationale	6
	2b	Specific objectives or hypotheses	6
**Methods**			
Trial design	3a	Description of trial design (such as parallel, factorial) including allocation ratio	8
	3b	Important changes to methods after trial commencement (such as eligibility criteria), with reasons	n.a.
Participants	4a	Eligibility criteria for participants	8
	4b	Settings and locations where the data were collected	8
Interventions	5	The interventions for each group with sufficient details to allow replication, including how and when they were actually administered	8
Outcomes	6a	Completely defined pre-specified primary and secondary outcome measures, including how and when they were assessed	9
	6b	Any changes to trial outcomes after the trial commenced, with reasons	n.a.
Sample size	7a	How sample size was determined	n.a.
	7b	When applicable, explanation of any interim analyses and stopping guidelines	n.a.
Randomisation:			
Sequence generation	8a	Method used to generate the random allocation sequence	n.a.
	8b	Type of randomisation; details of any restriction (such as blocking and block size)	8
Allocation concealment mechanism	9	Mechanism used to implement the random allocation sequence (such as sequentially numbered containers), describing any steps taken to conceal the sequence until interventions were assigned	n.a.
Implementation	10	Who generated the random allocation sequence, who enrolled participants, and who assigned participants to interventions	n.a.

Blinding	11a	If done, who was blinded after assignment to interventions (for example, participants, care providers, those assessing outcomes) and how	8
	11b	If relevant, description of the similarity of interventions	n.a.
Statistical methods	12a	Statistical methods used to compare groups for primary and secondary outcomes	9
	12b	Methods for additional analyses, such as subgroup analyses and adjusted analyses	9
**Results**			
Participant flow (a diagram is strongly recommended)	13a	For each group, the numbers of participants who were randomly assigned, received intended treatment, and were analysed for the primary outcome	11
	13b	For each group, losses and exclusions after randomisation, together with reasons	11
Recruitment	14a	Dates defining the periods of recruitment and follow-up	11
	14b	Why the trial ended or was stopped	n.a.
Baseline data	15	A table showing baseline demographic and clinical characteristics for each group	n.a.
Numbers analysed	16	For each group, number of participants (denominator) included in each analysis and whether the analysis was by original assigned groups	11
Outcomes and estimation	17a	For each primary and secondary outcome, results for each group, and the estimated effect size and its precision (such as 95% confidence interval)	11
	17b	For binary outcomes, presentation of both absolute and relative effect sizes is recommended	11
Ancillary analyses	18	Results of any other analyses performed, including subgroup analyses and adjusted analyses, distinguishing pre-specified from exploratory	12
Harms	19	All important harms or unintended effects in each group (for specific guidance see CONSORT for harms)	n.a.
**Discussion**			
Limitations	20	Trial limitations, addressing sources of potential bias, imprecision, and, if relevant, multiplicity of analyses	14
Generalisability	21	Generalisability (external validity, applicability) of the trial findings	13-14
Interpretation	22	Interpretation consistent with results, balancing benefits and harms, and considering other relevant evidence	14
**Other information**			
Registration	23	Registration number and name of trial registry	8
Protocol	24	Where the full trial protocol can be accessed, if available	8
Funding	25	Sources of funding and other support (such as supply of drugs), role of funders	18

*We strongly recommend reading this statement in conjunction with the CONSORT 2010 Explanation and Elaboration for important clarifications on all the items. If relevant, we also recommend reading CONSORT extensions for cluster randomised trials, non-inferiority and equivalence trials, non-pharmacological treatments, herbal interventions, and pragmatic trials. Additional extensions are forthcoming: for those and for up to date references relevant to this checklist, see www.consort-statement.org.
